# Supplementary material for: Anisotropic energy transfer in crystalline chromophore assemblies
Source: Nat Commun. 2018 Oct 18;9:4332. doi: 10.1038/s41467-018-06829-3 (PMC6193941; doi:10.1038/s41467-018-06829-3)
Supplement: Supplementary file 1 — Supplementary Information [file 41467_2018_6829_MOESM1_ESM.pdf]

Supplementary Information

Anisotropic energy transfer in crystalline chromophore assemblies

Haldar et al.

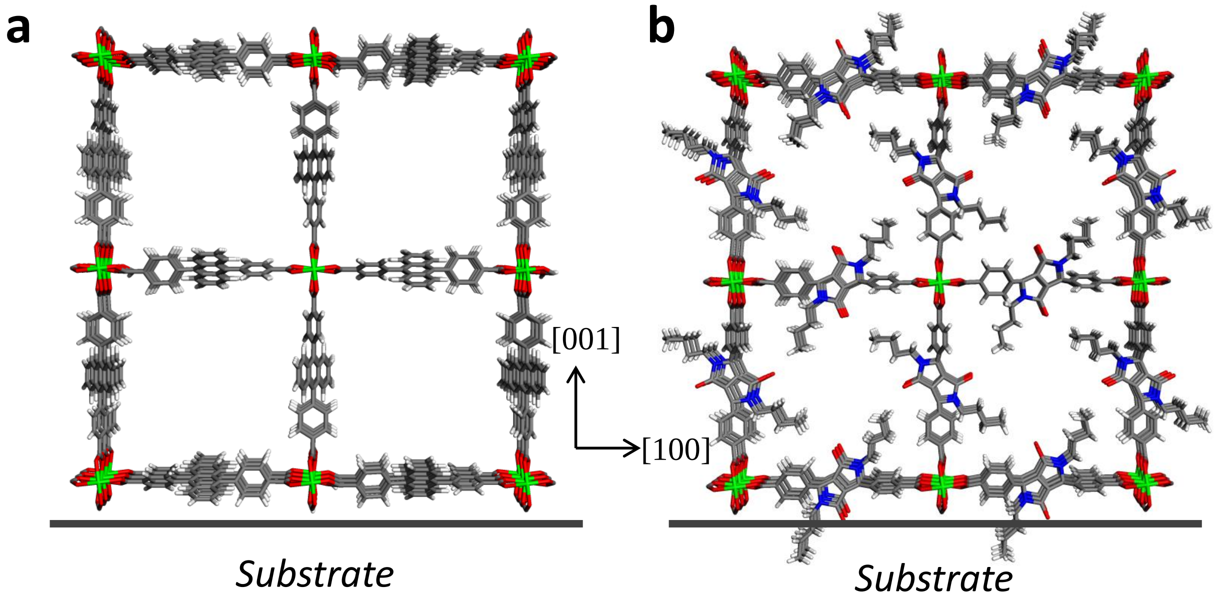

Supplementary Figure 1: **Structure of the SURMOFs.** View of the (**a**) Zn-ADB and (**b**) Zn-DPP SURMOF-2 structures along [010] direction.

a

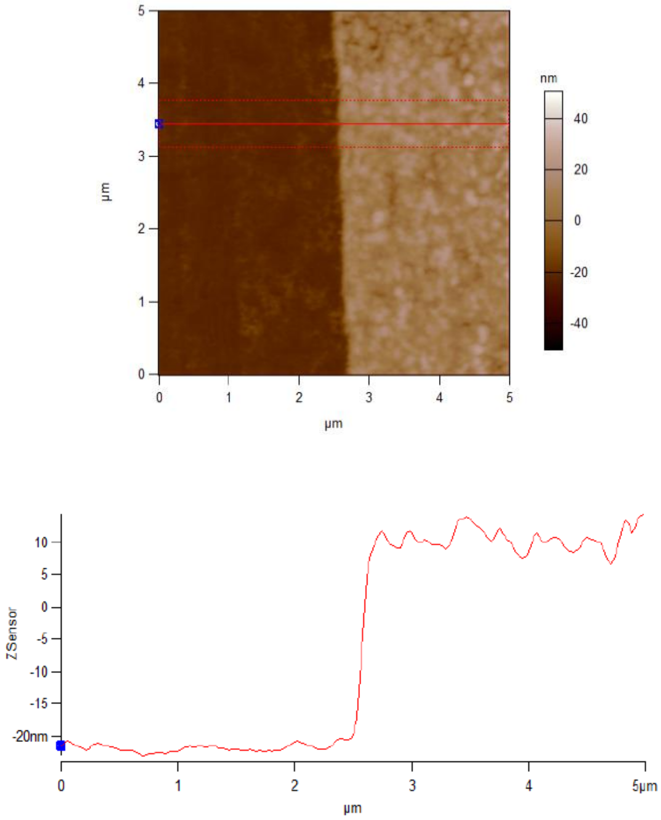

b

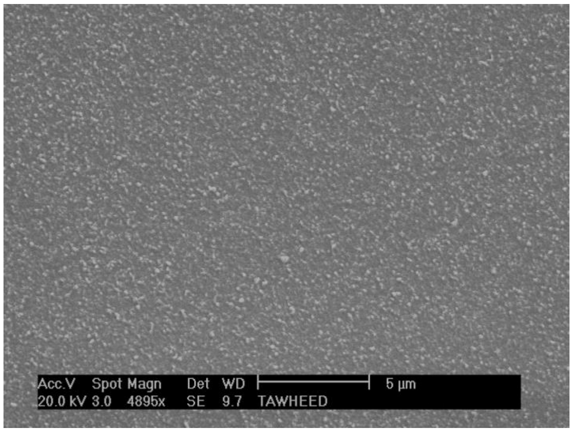

Supplementary Figure 2: **Thickness and morphology of the thin film.** (**a**) AFM image of Zn-ADB (2000 rpm) with a film thickness of ~ 35 nm after 40 cycle deposition by spin coating method. (**b**) SEM image of Zn-ADB SURMOF prepared using a 40 cycle deposition employing the spin coating method.

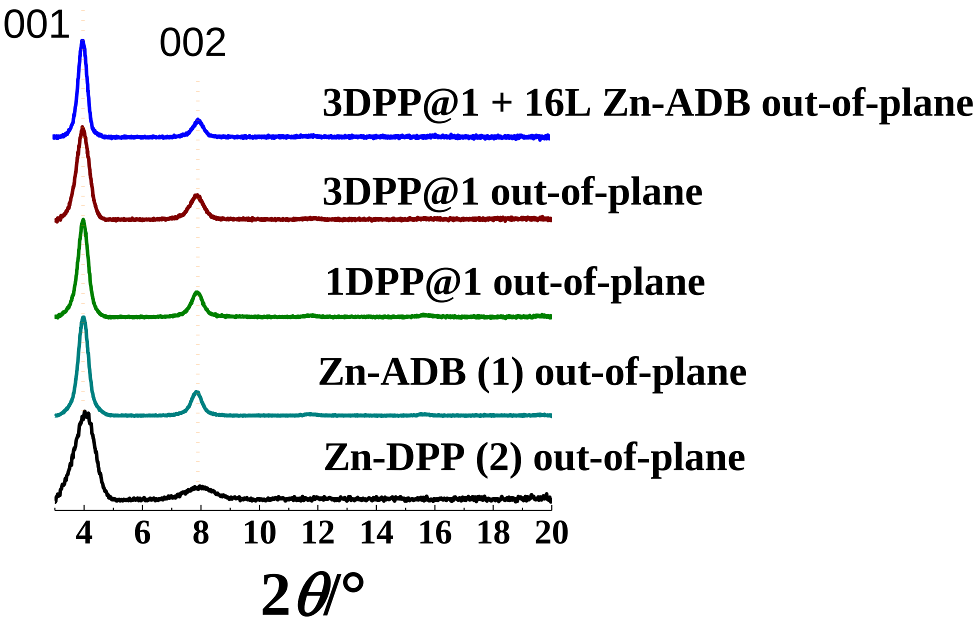

Supplementary Figure 3: **X-ray diffractions.** Out-of-plane XRD patterns of the pristine 1 and 2, and the mixed linker and heterostructured SURMOFs. The identical diffraction peaks for (001) and (002) planes suggests unchanged parent structure for all the SURMOFs.

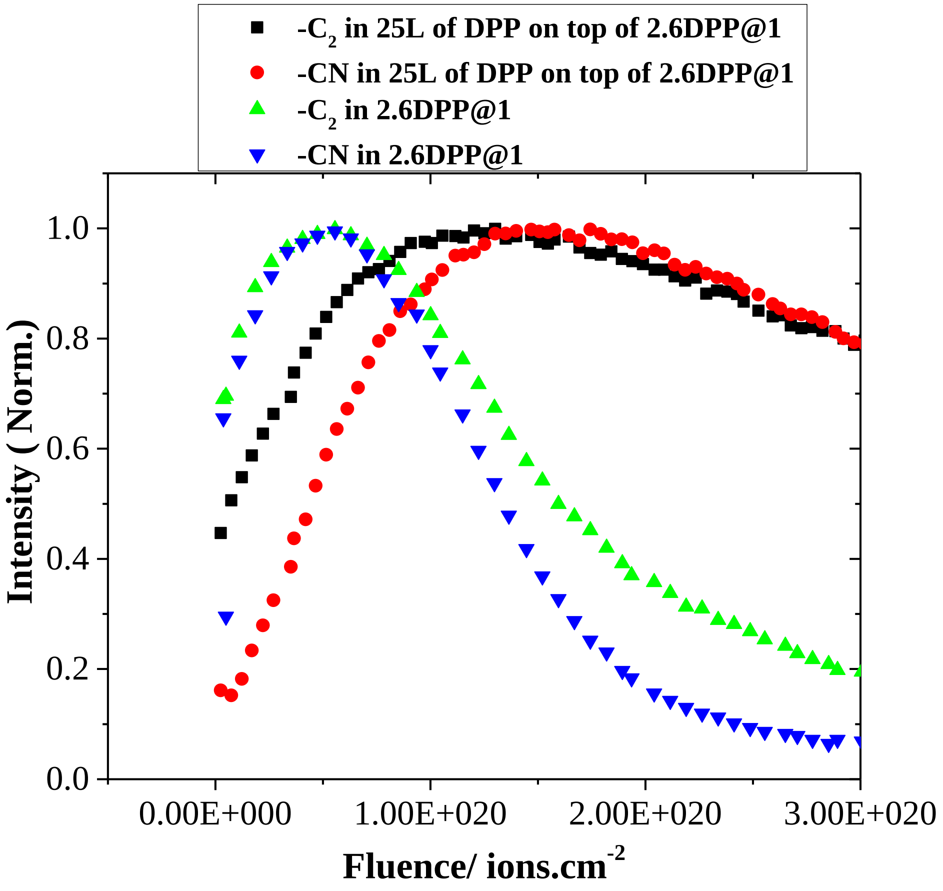

Supplementary Figure 4: **Analysis of the bilayer structure by ToF-SIMS.** ToF-SIMS depth profile of 2.6DPP@1 alone and 2.6DPP@1 as bottom layer and 25 cycle of 1 on top. The carbon content (-C2) and -CN represents the whole SURMOF structure and only the 2.6DPP@1, respectively. The delayed rise of -CN compared to -C2 hints top layer of 1 and bottom layer of 2.6DPP@1. Considering the thickness (~ 20 nm) of the top layer, i.e. pristine 1, such delayed rise of –CN signal is significant and suggests a distinct interface. In case of only 2.6DPP@1 –C2 and –CN intensity overlaps perfectly indicating homogeneously distributed DPP in 1.

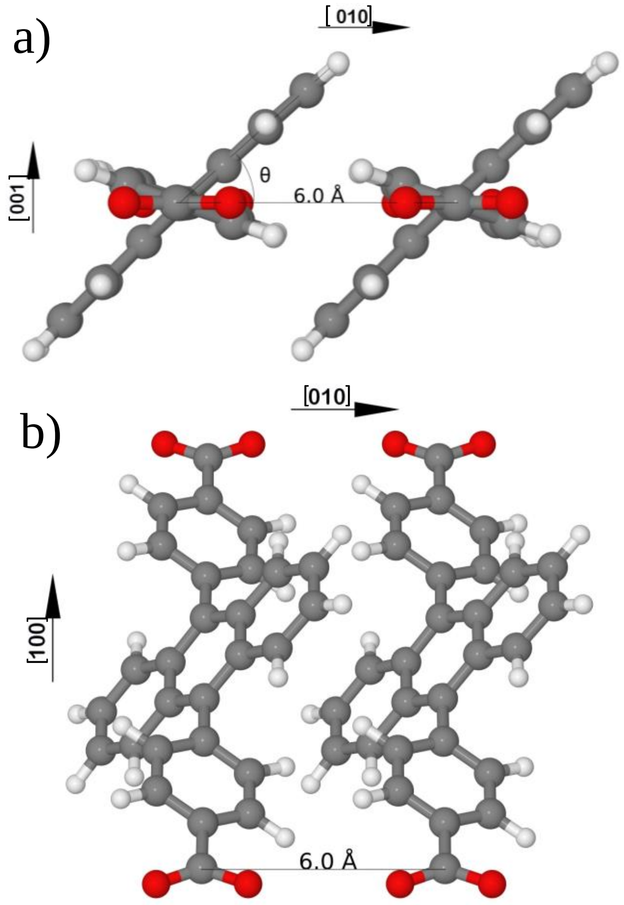

Supplementary Figure 5: Energy minimized structure. Energy optimized geometry of the ADB linkers employing optimized potential for liquid simulation (OPLS) force-field; (carried out considering the structural constrains in Zn-ADB SURMOF-2). (a) and (b) show the orientations of ADB linkers along two different directions, along [001] and [100], respectively.

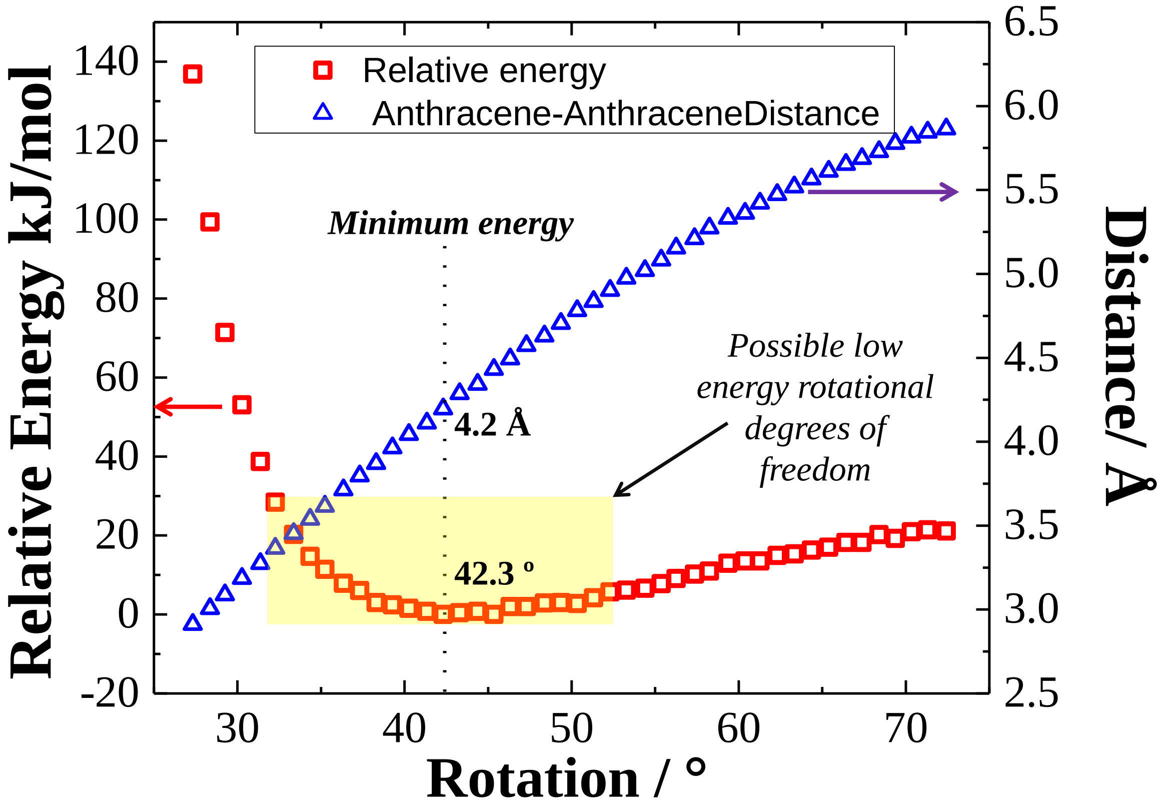

Supplementary Figure 6: **Rotation of the ADB linker in SURMOF.** Relative energy change and ADB-ADB distance plotted against the relative degree of rotation of the anthracene rings. The rotation of the anthracene is represented as θ in supplementary figure 5. The minimum energy geometry is shown in supplementary figure 5 and indicated here by the black dotted line. The yellow box shows the region, where the inter-anthracene distance falls below 3.8 Å, preferred to form excimer, with maximum energy requirement of 28 kJ/mol.

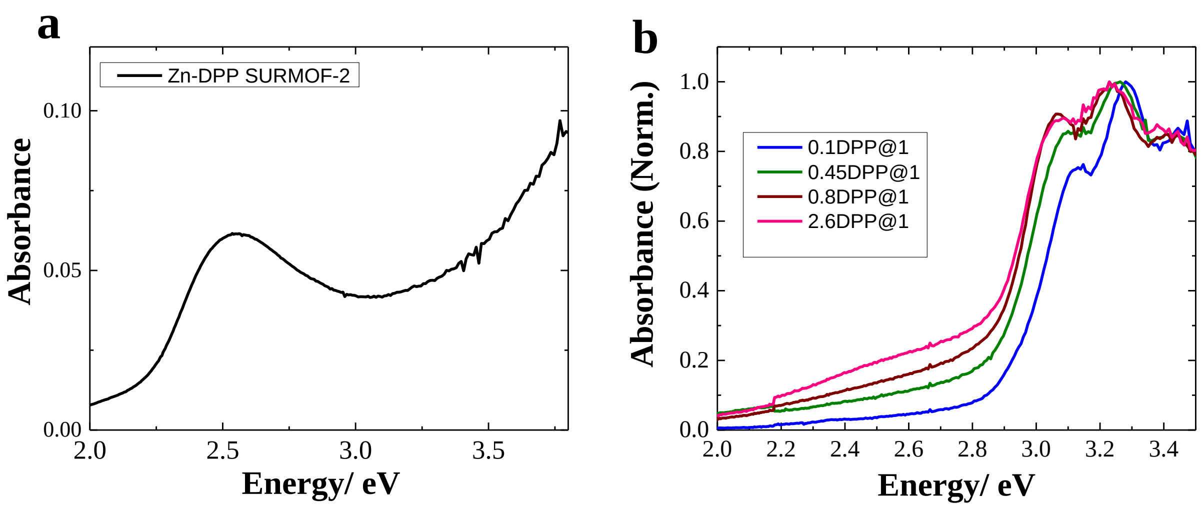

Supplementary Figure 7: **Absorption spectra.** (**a**) UV-Vis spectrum of Zn-DPP SURMOF-2. (**b**) UV-Vis spectra of 0.1DPP@1, 0.45DPP@1, 0.8DPP@1, and 2.6DPP@1 SURMOFs. With increasing concentration of DPP a broad band appears ~ 2.4-2.6 eV suggesting the presence of DPP in mixed-linker DA SURMOFs.

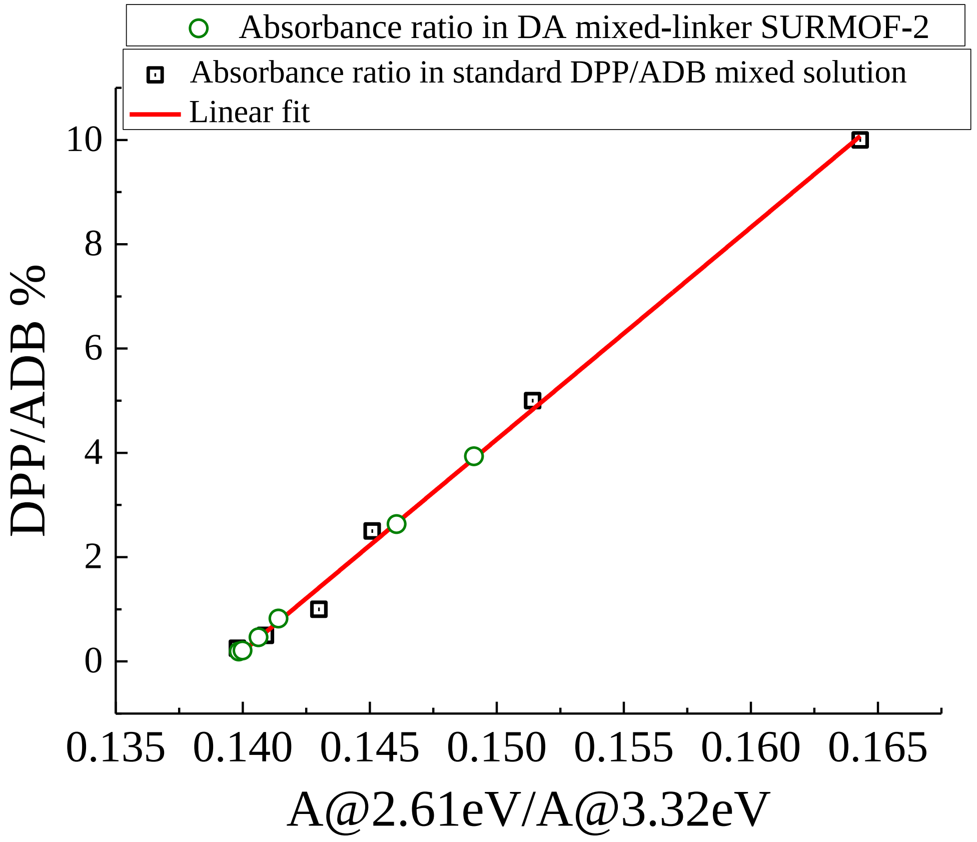

Supplementary Figure 8: Determination of the DPP/ADB ratio. To determine the DPP/ADB ratio in the DA mixed-linker SURMOF-2 structures, we have dissolved the structure using 10% acetic acid in ethanol and measured the absorption spectra. As a reference, absorption of a known ratio of DPP and ADB mixtures (in ethanol) has been measured. Ratio of absorption at 2.61 and 3.32 eV were plotted against the DPP/ADB % for the known mixture solutions (black square), and the linear fitted line (red) was used to identify the DPP concentration in the mixed-linker SURMOFs.

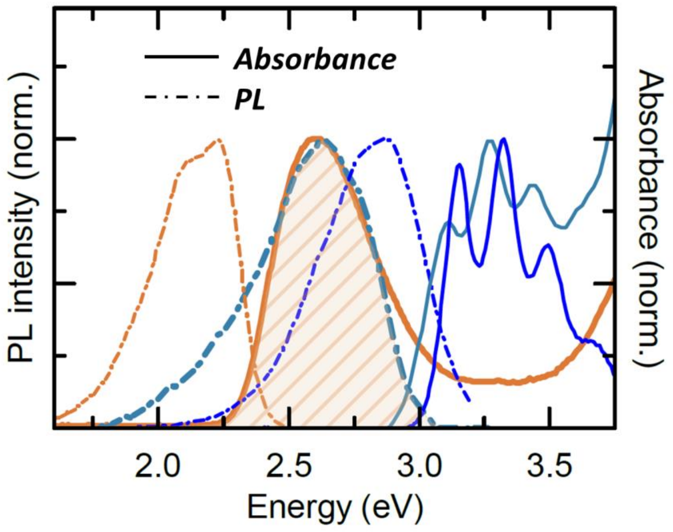

Supplementary Figure 9: PL and absorption spectra of the SURMOF. Absorption and PL spectra of ADB (blue), and DPP (orange) in ethanol (20 μM), and Zn-ADB (1) (cyan) at room temperature. The orange marked area shows the spectral overlap between the absorption of DPP and PL of 1.

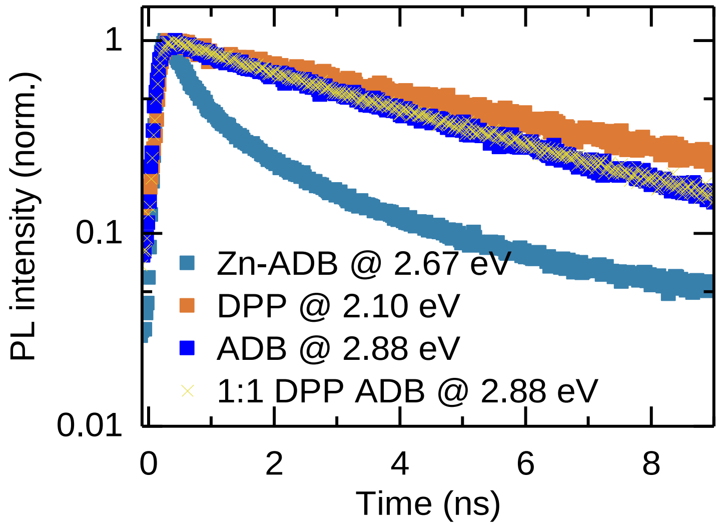

Supplementary Figure 10: **PL decay plots.** PL decay of the ADB and DPP linkers in ethanol, 1:1 mixture of ADB:DPP in ethanol and Zn-ADB (1) (E_{Ex} = 3.26 eV). The similar decay profile of ADB:DPP solution mixture and ADB solution suggests no energy transfer from ADB to DPP

in solution state. This could be due to no suitable interaction between the linkers in solution state or due to the insufficient spectral overlap.

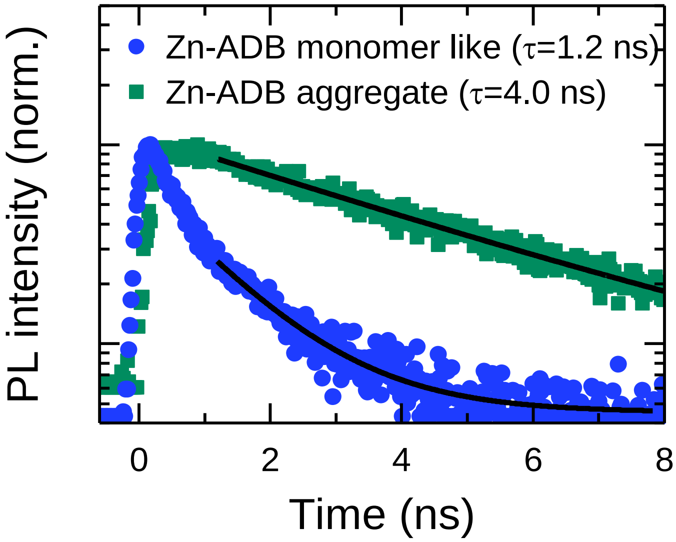

Supplementary Figure 11: **PL decay plots.** PL decay of PL_{Mon} and PL_{Exc} state in 1 (E_{Ex} = 3.26 eV).

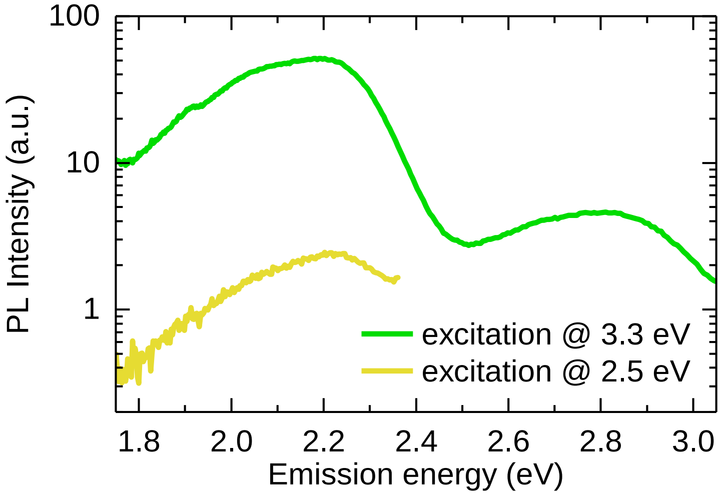

Supplementary Figure 12: **Excitation spectra of 1.** PL of 0.45@1 with excitation at 3.3 and 2.5 eV. Direct excitation of the DPP at 2.5 eV shows lower intensity, indicating the energy transfer process from 1 to DPP.

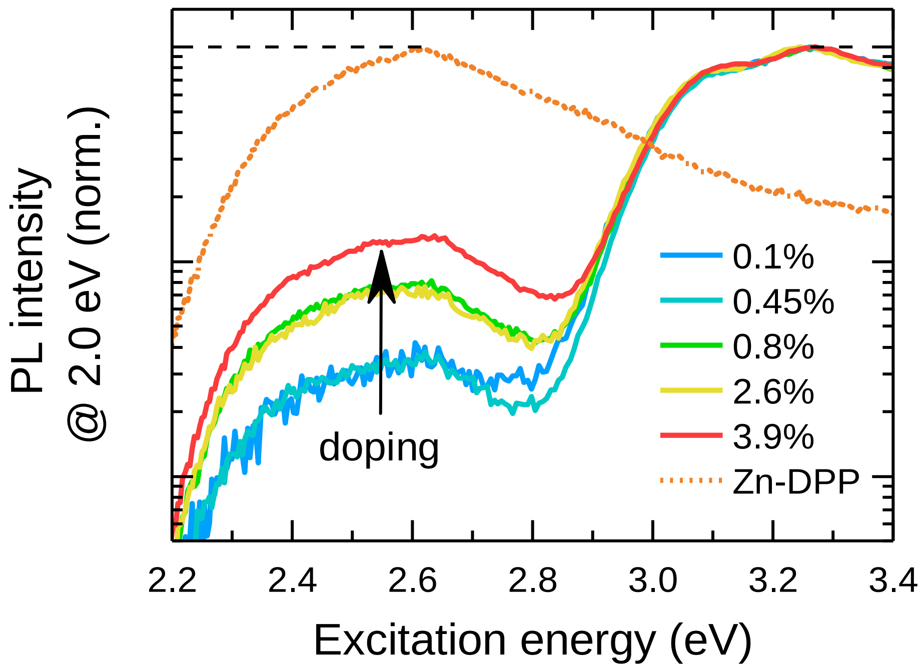

Supplementary Figure 13. **Excitation spectra of the mixed-linker SURMOFs.** Excitation spectra of the mixed-linker DA SURMOF-2 structures and pristine 1: The arrow indicates the change with higher % of DPP doping. The mixed-linker DA SURMOF-2 excitation spectra are normalized at donor excitation.

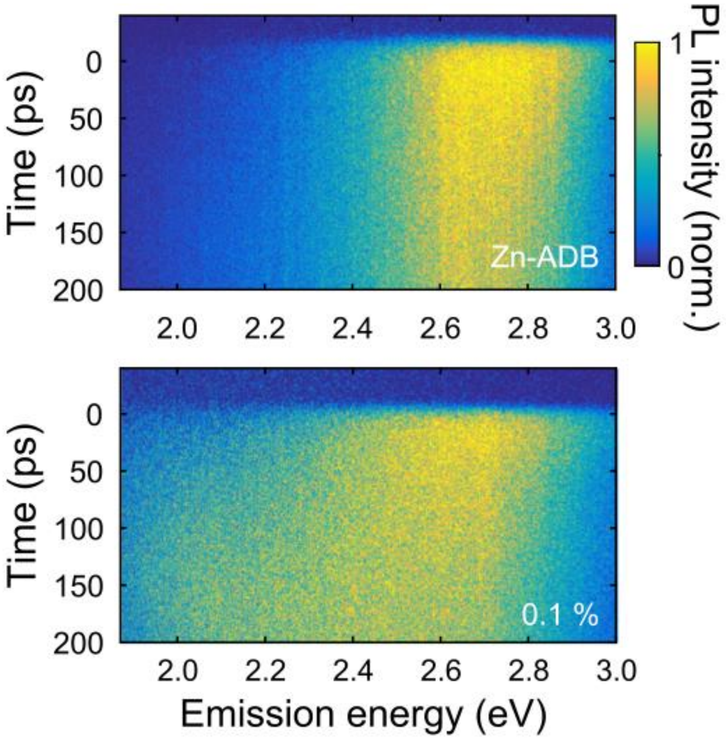

Supplementary Figure 14: **Streak camera image.** PL intensity as a function of energy and time obtained from streak camera system (E_{Ex} = 3.26 eV): upper panel is of 1, lower panel is of 0.8DPP@1. The lower panel image clearly exhibits the emergence of delayed acceptor PL, suggesting an energy transfer process.

Rate equations

**Zn-ADB (1)**	$k_{mon} \ll k^{1}{}_{\mathrm{T}}$
**1DPP@1**	$\eta_{S1\rightarrow Q} = \frac{k^{2}{}_{\mathrm{T}}}{k_{\mathrm{mon}}+k^{1}{}_{\mathrm{T}}+k^{2}{}_{\mathrm{T}}} = 0.4$<br>$\eta_{Exc\rightarrow Q} = \frac{k^{3}{}_{\mathrm{T}}}{k_{\mathrm{exc}}+k^{3}{}_{\mathrm{T}}} = 0.6$<br>$\eta_{S1\rightarrow Exc} = \frac{k^{1}{}_{\mathrm{T}}}{k_{\mathrm{mon}}+k^{1}{}_{\mathrm{T}}+k^{2}{}_{\mathrm{T}}} = 0.6$<br>$\eta_{\mathrm{total}} = \eta_{\mathrm{S1\rightarrow Exc}} \times \eta_{\mathrm{Exc\rightarrow Q}} + \eta_{\mathrm{S1\rightarrow Q}} = 0.76$

Supplementary Figure 15: **Rate equations of energy transfer.** Rate equations of the pristine and DA mixed-linker SURMOFs.

Supplementary Table 1: **PL Decay rate equations.** Decay rates of PL_{Exc} states (at 2.75 eV) in DA mixed-linker SURMOF-2 structures.

**DPP %**	**Measured Decay Rates 1/S**	k_{exc}	$k^{3}{}_{\mathrm{T}}$
0	2.5×10^{8}	2.5×10^{8}	---------------------
0.1	3.18×10^{8}		6.82×10^{7}
0.15	4.47×10^{8}		1.97×10^{8}
0.45	6.93×10^{8}		4.43×10^{8}
0.8	8.69×10^{8}		6.19×10^{8}
2.6	9.46×10^{8}		6.96×10^{8}
3.9	1.01×10^{9}		7.58×10^{8}

Supplementary Table 2: **PL rise rate equations:** Rise rates of PL_{Mon} states (at 1.98 eV) in DA mixed-linker SURMOF-2 structures.

DPP %	Measured Decay Rates 1/S	$k_{mon} + k^{1}_{T}$	k^{2}_{T}
0	1.21×10^{10}	1.21×10^{10}	---------------------
0.1	1.30×10^{10}		9.03×10^{8}
0.45	1.59×10^{10}		3.83×10^{8}
0.8	1.89×10^{10}		6.80×10^{8}
2.6	1.99×10^{10}		7.79×10^{8}
3.9	2.11×10^{10}		8.95×10^{8}

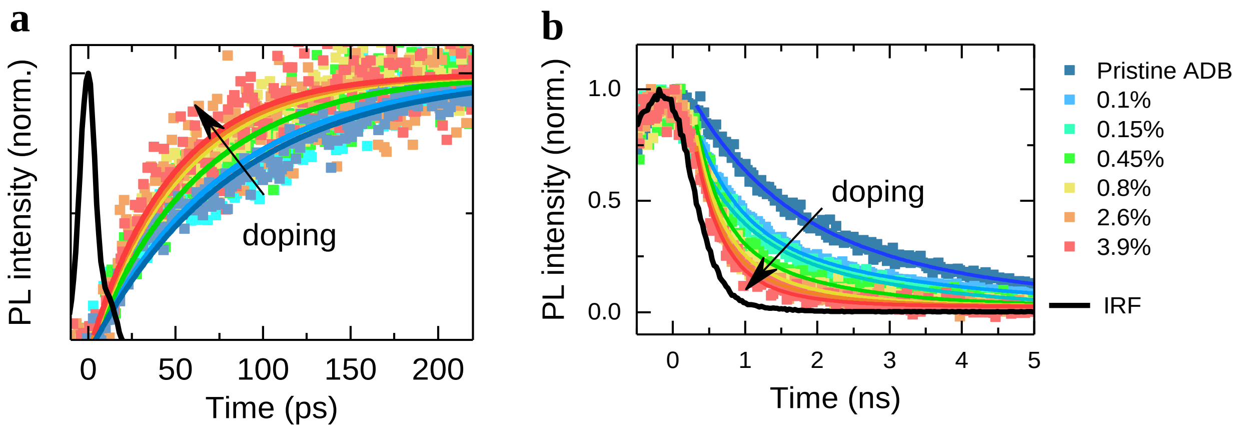

Supplementary Figure 16: **PL decay and rise kinetics.** (**a**) Rise-time of DPP emission (1.98 eV) as a function of the DPP concentrations in the doped SURMOF-2 structures. (**b**) PL decay of the PL_{Exc} state in 1 as a function of DPP concentrations in the doped SURMOF-2 structures. The black lines represent the instrument response. Figure 16a shows the rise time of the DPP emission as a function of the DPP fraction in the doped SURMOF-2 obtained from Streak camera measurements. To obtain this data, the PL was integrated over the 1.91-2.07 eV window.

We compare these rise times to that of the PL_{Exc} state PL in pristine 1. In the mixed-linker SURMOF-2, the rise time of the DPP emission is always faster than the rise time of the PL_{Exc} state emission in 1. This indicates that exciton arrives at the DPP after being transported by the PL_{Mon} population. In Figure 16b the lifetime of the PL_{Exc} species is shown as a function of the DPP concentration for the pristine 1 and doped SURMOF-2 structures. Here the lifetime is obtained using time-correlated single-photon counting (TCSPC) detecting ~ 2.6 eV photons. As the DPP concentration increases, the PL_{Exc} state lifetime decreases, confirming the mobility of the PL_{Exc} states.

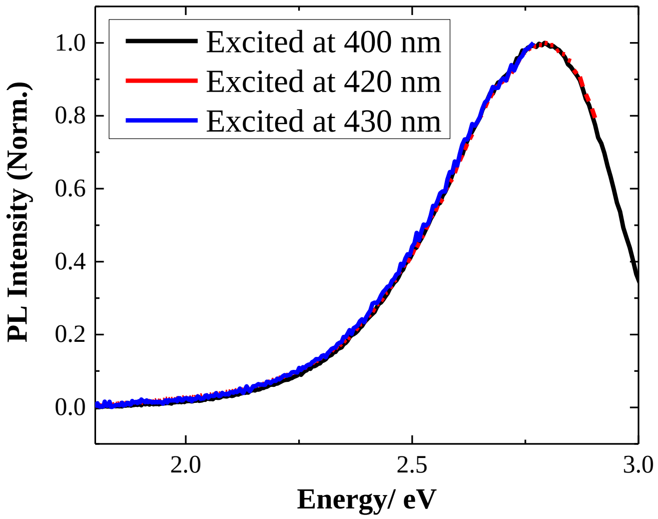

Supplementary Figure 17: **Excitation spectra of 1.** PL spectra of Zn-ADB upon excitation at 380, 400 and 420 nm show no shift in the PL, suggesting unchanged ratio of PL_{Mon}/PL_{Exc} state. This signifies that PL_{Exc} state does not absorb light and is created by the PL_{Mon} state.

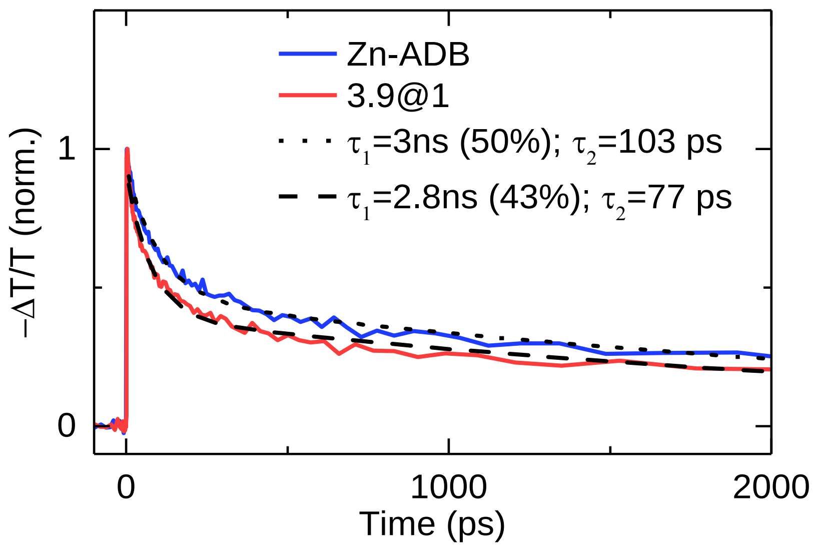

Supplementary Figure 18: **Transient absorption spectra of 1**. Shorter delay transient absorption decays of pristine Zn-ADB (1) and 3.9@1. No long lived component can be seen in 3.9@1, suggesting absence of any charge transfer (CT) state.

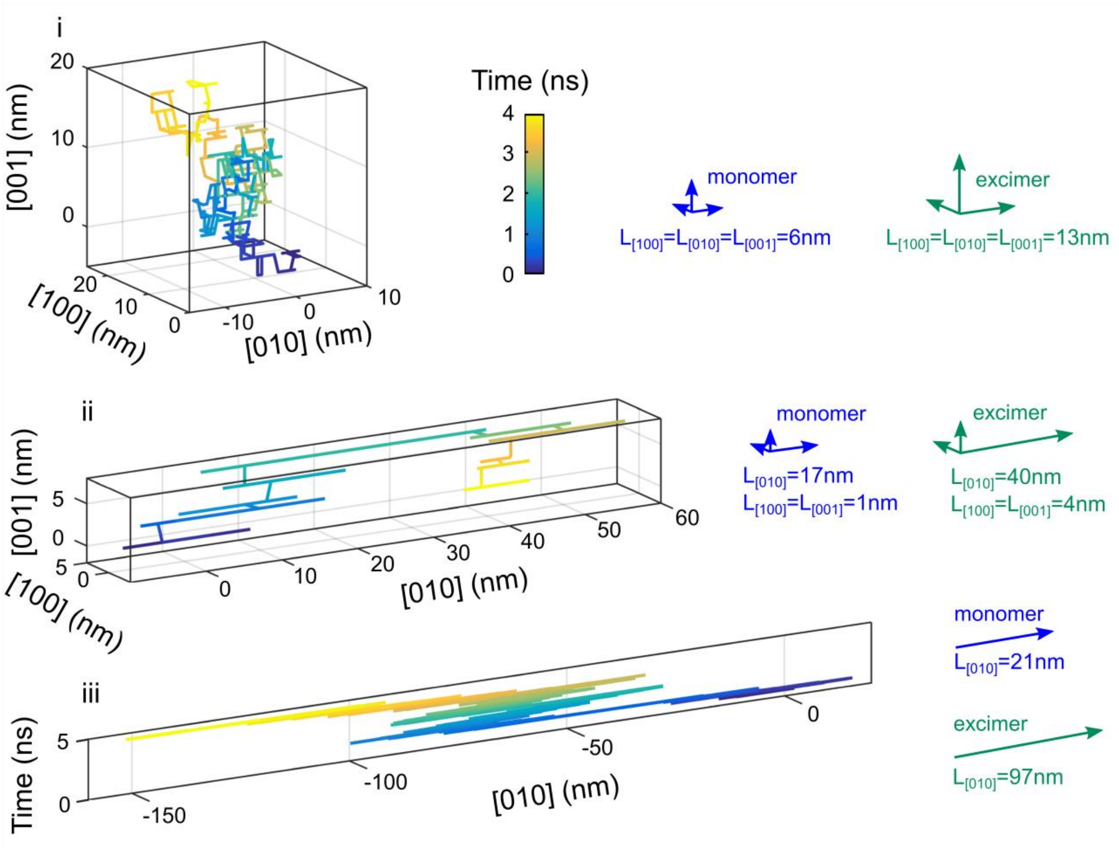

Supplementary Figure 19: Monte Carlo simulation. Representative Monte Carlo simulations of a PL_{Exc} state's motion to 330 unique chromophore sites considering (**a**) isotropic 3D diffusion, (**b**) anisotropic 3D diffusion rates scaling as $1/R^6$ with hopping distance in the tetragonal lattice leading to a factor of 1000 faster transfer in the x-direction than in the y- and z-directions, and (**c**) 1D diffusion in which only transfer in the x-direction is considered. The average displacement of the excited-state after hopping to a given number of unique sites (*i.e.* the diffusion length of the excited state) varies strongly with the dimensionality of the diffusion. With increasing dimensionality of the diffusion, the average maximum displacement from the excited-state's original position decreases. This is illustrated in the Monte Carlo simulations for excited-state motion shown in Figure 19. These simulations are not meant to reproduce the photophysics of this system with full accuracy, but rather illustrate how the effective diffusion length for a particle that visits a set number of sites varies with the anisotropy of the transfer rates in the different directions. In Figure 19a the motion is illustrated for the case wherein the product of the rate constant for a hop and the distance hopped in all the three directions are taken to be constant (Here, [010] direction, represents the stacking direction of the ADB linkers). This

results in an isotropic diffusion length. For the monomer (70 unique sites visited) this corresponds to a root-mean-square displacement of 2 linkers in [100] and [001]-directions and 7 linkers in [010]-direction. For the excimer this would result in a diffusion length of 5 linkers in [100] and [001]-directions and 21 linkers in [010]-direction. With the geometry of our system, this corresponds to diffusion lengths of the monomer and excimer of 6 and 13 nm, respectively. We note that for the simulation, an excited-state is allowed to hop until it reaches the desired number of unique sites have been visited (either 70 or 330 for the PL_{Mon} or PL_{Exc} states, respectively). The trajectory for a single Monte Carlo simulation of a PL_{Exc} state is shown, with the time axis estimated by the time per hop given by the total number of hops and the measured PL_{Exc} state lifetime. The root-mean-square maximum displacement in each lattice direction and the total maximum displacement (diffusion length) are calculated by averaging over 1000 simulations. In Figure 19b, we examine the situation wherein the diffusion is still 3D, but with anisotropic rate constants. Due to the $1/R^6$ dependence of FRET, we can estimate form the geometry of our system that the hops in the inter-sheet [010]-direction are $\gtrsim 1000$ times faster than in the [100] and [001]-directions. Here, for this simple illustration, we neglect differences in the inter-chromophore coupling factor, which would also favor transport in the [010]-direction. Simulations for such anisotropic 3D diffusion lead to trajectories illustrated for a PL_{Exc} state in Figure 19b. Here, the motion in the [010]- direction is clearly favored. In this case, averaging over 1000 Monte Carlo simulations leads to a diffusion length for the PL_{Mon} and PL_{Exc} state of 28 and 66 linkers, respectively along [010]-direction. Provided the geometry of our system, those corresponds to diffusion lengths of 17 and 40 nm, respectively. However, the transport lengths are anisotropic with an average maximum displacement in the [100] and [001]-directions 1 and 4 nm for PL_{Mon} and PL_{Exc} states, respectively. Finally, we illustrate the extreme case of purely 1D diffusion, where only hops in the [010]-direction are considered possible (Figure 19c). In this case, the root-mean-square displacements for the excited-states are considerably longer and approximately equal to half of the number of unique sites visited: 35 and 161 linkers for the PL_{Mon} and PL_{Exc} state, respectively (corresponding to 21 and 97 nm in the given geometry). In summary, the mixed linker approach allows us to determine that both the excited states are mobile, and that they visit 70 (PL_{Mon}) and 330 (PL_{Exc}) unique linkers within their lifetimes. However, we see that the diffusion length of those two excited states depends on the anisotropy of their hopping rates.

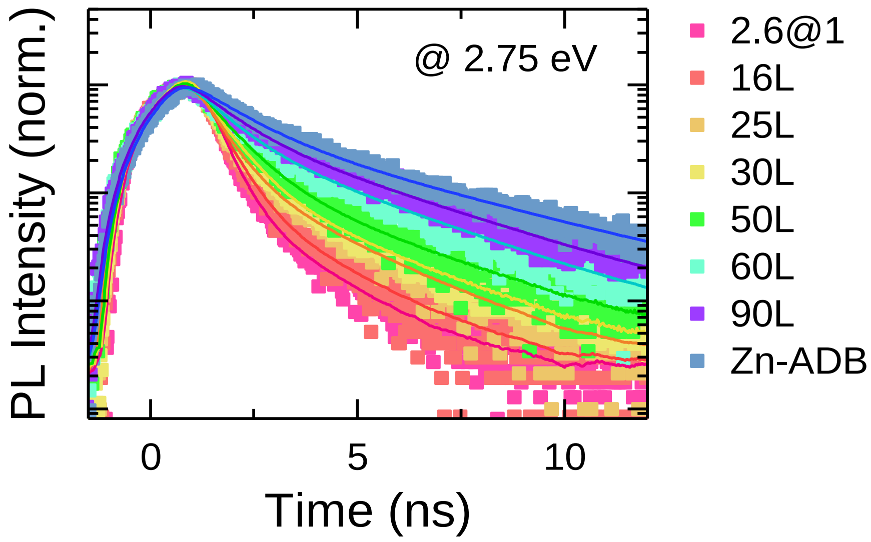

Supplementary Figure 20: PL decay profiles of the bilayer SURMOFs. PL transients of the donor excitons at 2.75 eV for various thicknesses of the donor layer on top of the 2.6DPP@1. Each transient is accompanied by a biexponential reconvolution fit.

Supplementary Table 3. **PL Decay rate equations.** Decay rates of PL_{Mon} and PL_{Exc} states in heterostructured SURMOF-2; (amplitude of PL_{Exc} state = (1 - amplitude of PL_{Mon})).

**No. of Donor layer deposition cycle on top of 2.6@1**	**Decay Rates of PL_{Exc} state**	**Decay Rates of PL_{Mon} state**	**Amplitude PL_{Mon} state**
0	8.13×10^{8}	3.59×10^{9}	0.993
16	5.56×10^{8}	2.15×10^{9}	0.979
25	4.47×10^{8}	1.98×10^{9}	0.962
30	3.95×10^{8}	1.59×10^{9}	0.936
50	3.37×10^{8}	1.30×10^{9}	0.926
60	3.14×10^{8}	1.25×10^{9}	0.849
90	2.91×10^{8}	1.12×10^{9}	0.8002
**Zn-ADB**	2.43×10^{8}	8.61×10^{8}	0.7678

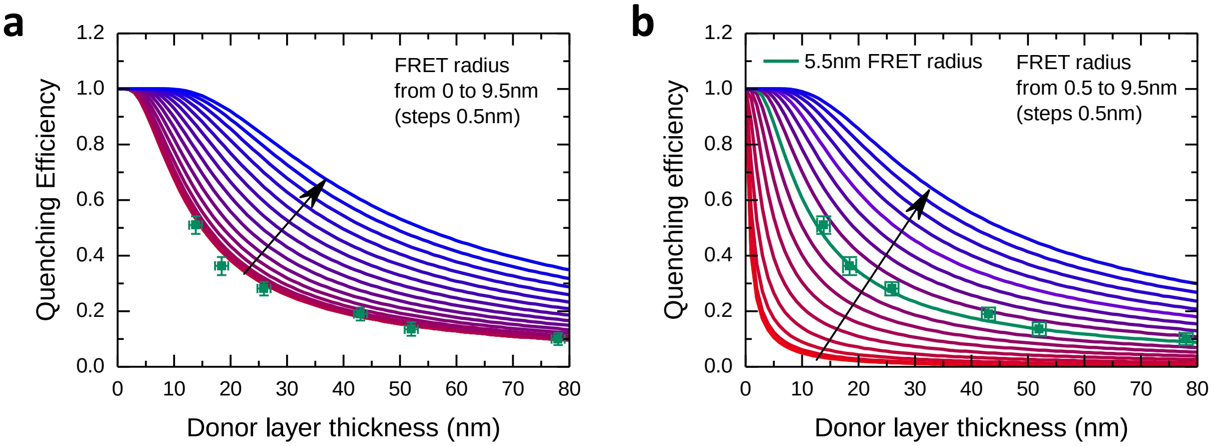

Supplementary Figure 21: **Simulated and experimental quenching efficiencies.** PL$_{Exc}$ sate quenching efficiencies of the top layer only (green symbols) and simulated quenching efficiencies plotted against the donor layer thickness; in (a) the simulation considers a 13 nm diffusion radius and varying FRET radius, (b) no diffusion but only varying FRET radius. The experimental data fits well with FRET radius of 5.5 nm, having no diffusion, suggesting an anisotropic diffusion model. The error bars are determined by (for y-axis) propagating the uncertainties of the fits in Supplementary Fig. 16 and 20, and (for x-axis) showing the surface roughness of the film.

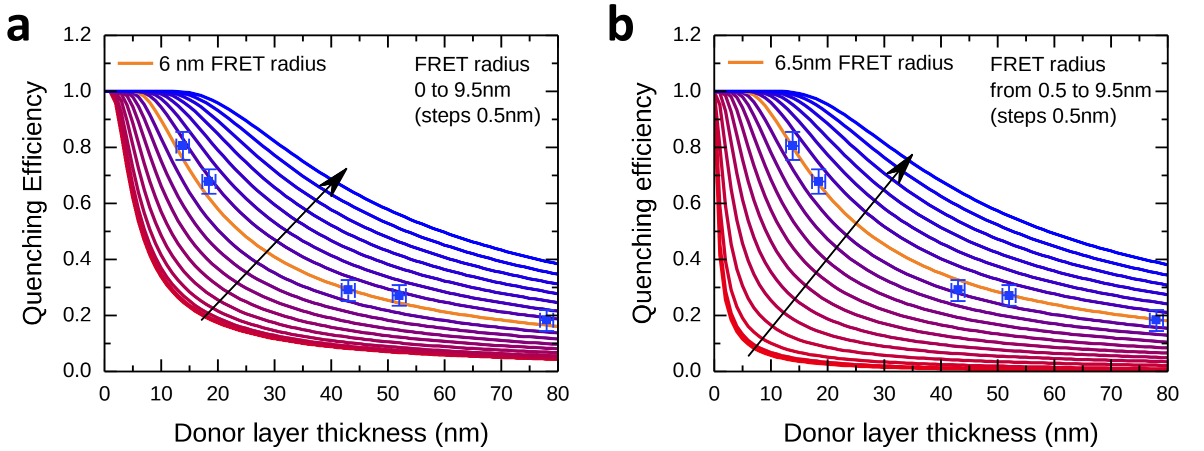

Supplementary Figure 22: **Simulated and experimental quenching efficiencies.** PL$_{Mon}$ sate quenching efficiencies of the top layer only (blue symbols) and simulated quenching efficiencies plotted against the donor layer thickness; in (a) the simulation considers a 6 nm diffusion radius and varying FRET radius, (b) no diffusion but only varying FRET radius. The experimental data

fits well with i) 6 nm diffusion radius and FRET radius of 6 nm, or ii) only 6.5 nm diffusion radius, having no diffusion. The error bars are determined by (for y-axis) propagating the uncertainties of the fits in Supplementary Fig. 16 and 20, and (for x-axis) showing the surface roughness of the film.

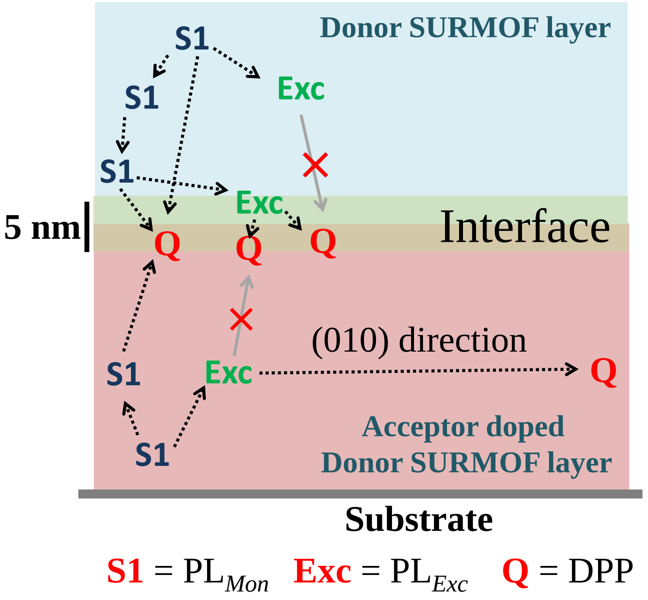

Supplementary Figure 23: **Schematic of exciton dynamics.** Exciton dynamics and energy transfer paths in the bilayer-SURMOF-2 structure.

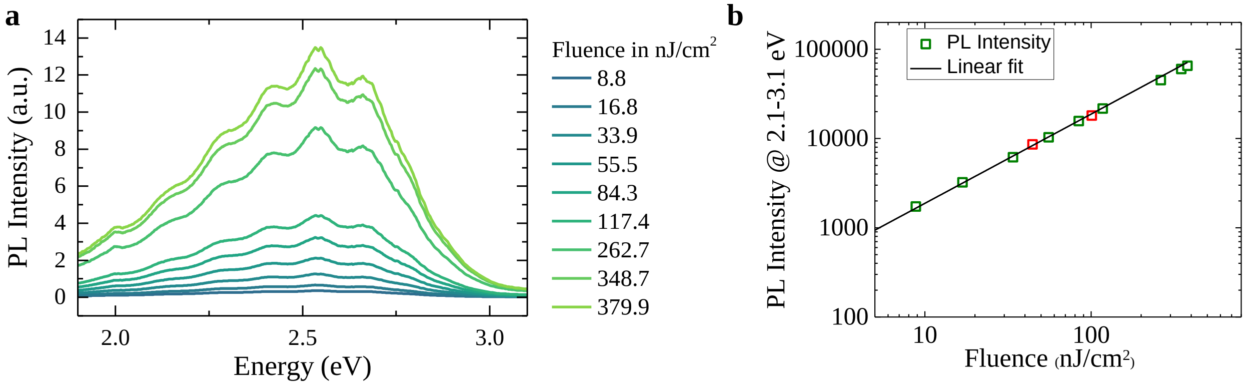

Supplementary Figure 24: **Fluence dependent PL.** (**a**) Fluence-dependent PL spectra in the range of 8.8-380 nJ/cm^2. (**b**) Integrated PL intensity vs fluence plot showing a linear dependence, which strongly indicates absence of annihilation processes. (Green box= low to high fluence; Red box= high to low fluence).

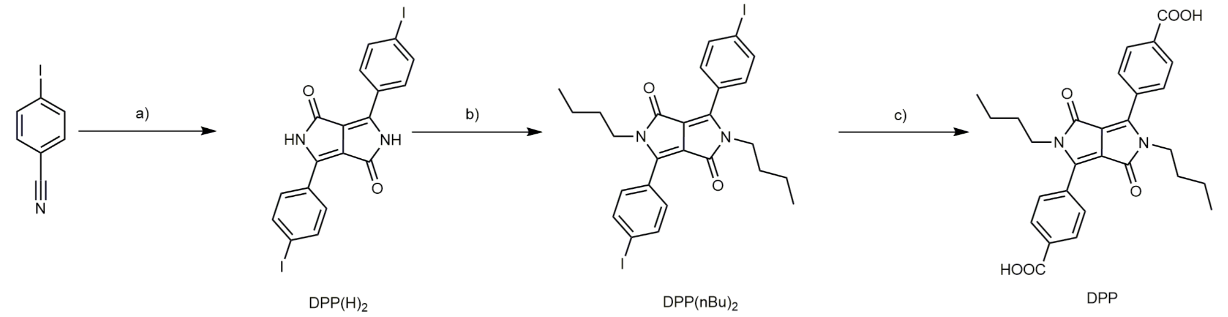

Supplementary Figure 25: **DPP synthesis scheme.** Synthesis of 2,5-bis(butyl)-3,6-bis(4-carboxylicphneyl-4-yl)-2,5-dihydropyrrolo[3,4-*c*]pyrrole-1,4-dione (DPP). a) Diethylsuccinate, (*t*-amylalcohol), Na^0, $FeCl_3$, 90°C, o/n, 57%. b) nBuI, *t*BuOK,(NMP), 60°C, o/n, 44%. c) $Pd(OAc)_2$, XantPhos, DMAP,$Co_2(CO)_8$, (toluene/H_2O), 90°C, o/n, 28%.

Supplementary Method

Materials. Zinc acetate dihydrate was purchased from Merck Millipore. 16-mercaptohexadecanoic acid (MHDA, 97%), was purchased from Sigma-Aldrich (Germany). Absolute ethanol was purchased from VWR (Germany).

Substrates. The silicon substrates with a [100] orientation are from Silicon Sense (US). The quartz glasses are from Alfa Aesar. These substrates were treated with plasma (Diener Plasma) under O_2 (50 sccm) for 30 min to remove the impurities and generated a surface with hydroxyl groups.

Synthesis of DPP(H)$_2$. A catalytic amount of $FeCl_3$ was dissolved in t-amyl alcohol (40 mL), small cubes of solid sodium were added (2.02 g, 88 mmol, 5 eq) and the mixture was heated at 90 °C for 40 min. A suspension of 4-iodobenzonitrile (10 g, 44 mmol, 2.5 eq) in t-amyl alcohol (25 mL) was added, followed by a dropwise addition of diethyl succinate (3.04 g, 17.5 mmol), the resulting red solution was heated at 90 °C overnight. The mixture was cooled at room temperature and acetic acid (10 mL) was added dropwise under vigorous stirring. After complete addition the red slurry was stirred at 110 °C for 30 minutes and then cooled at room temperature,

the resulting precipitate was recovered by filtration and washed with water and methanol to afford the desired compound as a dark powder. (5.40 g, 57%)

Due to its high insolubility in common organic solvents, **DPP(H)$_2$** was used without further purification.

Synthesis of DPP(nBu)$_2$. In a flame-dried schlenck tube, **DPP(H)$_2$** (0.1 g, 0.185 mmol) and potassium *tert*-butanolate (0.050 g, 4.44 mmol, 2.4 eq) were dissolved in dry N-methylpyrrolidone (10 mL) the resulting dark red solution was heated at 60 °C, upon heating the solution turned blue. 1-iodobutane (0.140 g, 0.76 mmol, 4.1 eq) was added and the solution was heated at 60 °C for 24h. The resulting bright orange solution was cooled at room temperature and toluene was added. The organic phase was washed three times with water and brine, dried onto $MgSO_4$ and concentrated *in vacuo*, the resulting orange residue was taken in methanol and the resulting red precipitate was removed by filtration, concentration of the orange liquors afforded the desired compound as an orange powder. (0.053 g, 44%) NMR (^{1}H, $CDCl_3$, 300 MHz) δ (ppm) 7.88 (d, 4H, *J* = 8.73 Hz), 7.55 (d, 4H, *J* = 8.73 Hz), 3.73 (t, 4H, *J* = 7.60 Hz), 1.55 (m, 2H), 1.25 (m, 6H), 0.85 (t, 6H, *J* = 7.29 Hz).NMR (^{13}C, $CDCl_3$, 300 MHz) δ (ppm) 162.518, 153.474, 147.731, 138.255, 130.072, 127.344, 98.057, 41.635, 31.728, 19.956, 13.639.
HRMS (ESI+) $[M+Na]^+$ *m/z* 653.0166 found, 653.0162 calc.

Synthesis of DPP. In a sealable tube, **DPP(nBu)$_2$** (0.053 g, 0.081 mmol), Pd acetate (5 mol%), XantPhos (10 mol%) and dimethylaminopyridine (0.012 g, 0.081 mmol, 1 eq) were dissolved in a mixture of toluene (7 mL) and water (3 mL). Cobalt carbonyl (0.011 g, 0.032 mmol, 0.33 eq) was added and the tube was sealed immediately. The biphasic mixture was heated at 90 °C under vigorous stirring overnight. The mixture was cooled to room temperature and was poured onto ethyl acetate, the organic phase was washed with an aqueous citric acid solution (10 w%), water and brine, dried onto $MgSO_4$ and concentrated under vacuum, the resulting red powder was washed with CH_2Cl_2 to afford the desired product. (0.011 g, 28%) NMR (^{1}H, THF-d*8*, 300 MHz) δ (ppm) 8.16 (d, 4H, *J* = 8.43 Hz), 7.99 (d, 4H, *J* = 8.43 Hz), 3.83 (t, 4H, *J* = 7.26 Hz), 1.50 (m, 4H), 1.24 (m, 4H), 0.82 (t, 6H, *J* = 7.40 Hz)
